# Bilateral giant myelolipomas in a female patient with untreated 21-hydroxylase deficiency

**DOI:** 10.1210/jcemcr/luag176

**Published:** 2026-07-27

**Authors:** Ann-Christin Welp, Matthias K Auer, Lea Tschaidse, Petra Zimmermann, Till Braunschweig, Nicole Reisch

**Affiliations:** Medizinische Klinik und Poliklinik IV, Klinikum der Universität München, Munich 80336, Germany; Medizinische Klinik und Poliklinik IV, Klinikum der Universität München, Munich 80336, Germany; Medizinische Klinik und Poliklinik IV, Klinikum der Universität München, Munich 80336, Germany; Klinik für Allgemein-, Viszeral- und Transplantationschirurgie, Klinikum der Universität München, Munich 80336, Germany; Pathologisches Institut, Klinikum der Universität München, Munich 80336, Germany; Medizinische Klinik und Poliklinik IV, Klinikum der Universität München, Munich 80336, Germany

**Keywords:** congenital adrenal hyperplasia, androgen excess, adrenal hyperplasia, adrenal

## Abstract

Adrenal myelolipomas are benign neoplasms of the adrenal gland composed of mature adrenal and adipose tissue as well as myeloid elements. Although infrequent in the general population, their prevalence is markedly increased in patients with poorly controlled classical congenital adrenal hyperplasia (CAH), most likely attributable to chronic ACTH-mediated adrenal stimulation. We report the case of a 59-year-old female with a history of classic CAH due to 21-hydroxylase deficiency who did not receive treatment for over 30 years and had developed bilateral giant myelolipomas (left side: 24.5 cm × 20.5 cm × 9.7 cm; right side: 14.5 cm × 11.6 cm × 6.5 cm), with the left-sided myelolipoma displacing abdominal organs. The prolonged excess of adrenal androgens resulted in hirsutism, progressive hair loss, secondary amenorrhea persisting for over 30 years, and a deepening of the voice. Bilateral adrenalectomy was performed; postoperatively, the patient received glucocorticoid and mineralocorticoid replacement. Her hyperandrogenemia resolved, and symptoms of hyperandrogenism improved gradually. This case illustrates the necessity of long-term hormonal replacement and regular follow-up in patients with CAH, as well as the multidisciplinary approach to prevent or treat long-term complications such as myelolipomas.

## Introduction

Congenital adrenal hyperplasia (CAH) is an autosomal recessive disorder characterized by impaired synthesis of adrenal glucocorticoids, leading to cortisol and aldosterone deficiency. Over 95% of cases result from 21-hydroxylase deficiency (21-OHD) due to pathogenic variation in the *CYP21A2* gene. CAH is classified into the classic form—comprising salt-wasting and simple virilizing types—and the nonclassic form [1, 2].

Loss of negative feedback in 21-OHD leads to chronic ACTH hypersecretion, driving adrenal hyperplasia and adrenal androgen excess [3, 4]. While ACTH-induced adrenal growth is well recognized, its role in tumor development is unclear [5]. Imaging studies in CAH are limited, and routine screening for adrenal masses is not recommended [6]. On the other hand, reported adrenal adenoma prevalence ranges from 11% to 83%, with myelolipomas as frequent as 37% of adrenal tumors in CAH [5, 7].

Adrenal myelolipomas are benign cortical tumors containing adipose and hematopoietic elements [8]. Once considered rare, their detection has increased with widespread abdominal imaging [9]. Adrenal tumors are found in up to 15% of older adults but remain uncommon below 40 years, most being nonfunctioning adenomas [5]. A meta-analysis found that 5.9% of patients with adrenal incidentalomas had a biochemical diagnosis consistent with CAH, and 0.8% had genetically confirmed CAH [10]. Screening with 17-hydroxyprogesterone (17-OHP) therefore is advised in patients with bilateral adrenal incidentalomas [11].

We report a female patient with classic CAH due to 21-OHD who remained untreated for over 30 years. This caused chronic adrenal hyperandrogenemia resulting in severe virilization and the development of bilateral giant myelolipomas. This case illustrates the long-term consequences of untreated 21-OHD and the importance of lifelong vigilant endocrine multidisciplinary management.

## Case presentation

We present a 59-year-old woman who was clinically and biochemically diagnosed with 21-OHD at birth. Subsequent genetic analysis of the *CYP21A2* gene confirmed c.293-13C > G heterozygously as well as a heterozygous deletion, resulting in about 1% residual enzyme activity in vitro [1]. During childhood and adolescence, cortisol and mineralocorticoids were replaced, and pubertal development was typical. From the age of 25 years, the glucocorticoid and mineralocorticoid replacement was discontinued by the patient, leading to secondary amenorrhea, voice deepening, hair loss, and substantial hirsutism. Except for severe virilization, the patient was not compromised in any daily activities; in particular, she did not experience any fatigue even though she was physically very active. She never followed any sick rules with intake or dose increase of glucocorticoid, nor did she experience any adrenal crises. Because of severe virilization, glucocorticoid and mineralocorticoid replacement were restarted at the age of 56 years, at a daily dose of 20 mg hydrocortisone (10 mg twice per day = 12.8 mg/m^2^/day) and 0.05 mg fludrocortisone without any therapeutic efficacy (ie, all symptoms described previously remained the same).

During a routine physical examination at the age of 59 years, the patient's general practitioner palpated a left-sided abdominal mass, which prompted a referral to the Department of Visceral Surgery and the Department of Endocrinology at the LMU Klinikum München due to the patient's known diagnosis of CAH.

## Diagnostic assessment

On initial presentation in our outpatient clinic for endocrinology, the patient was 59 years old, with height 160 cm, weight 54.9 kg (body mass index 21.5 kg/m^2^), and blood pressure 137/79 mmHg while seated. She was in good general condition, without abdominal pain. She presented with clinical signs of hyperandrogenemia, such as hirsutism (requiring daily shaving of facial hair), severe hair loss on the scalp (resulting in wearing a wig), a deep voice, and secondary amenorrhea since the age of 25 years. She had no children, no pregnancies, and never attempted to get pregnant. At a total daily hydrocortisone dosage of 20 mg (10 mg twice per day = 12.8 mg/m^2^/day), biochemically hyperandrogenemia was confirmed with substantially elevated total testosterone of 350 ng/dL (12.1 nmol/L; reference range [RR], 26-122 ng/dL [0.9-4.2 nmol/L]) and androstenedione of 1310 ng/dL (45.9 nmol/L; RR, <210 ng/dL [<7.4 nmol/L]) (Table 1). Steroid 17-OHP was massively increased, both in serum (3600 ng/dL [108.9 nmol/L]; RR, 30-270 ng/dL [1.0-8.2 nmol/L]) and saliva (before intake of the morning medication; 1178.3 pg/mL [3567.6 pmol/L]; RR, 22-84.8 pg/mL [66.6-256.6 pmol/L]; target range <254.4 pg/mL [769.5 pmol/L]).

**Table 1:** Hormonal concentrations, as well as additional parameters reflecting adrenal function and metabolic status, before and 2 weeks after adrenalectomy

Parameter	Reference range	Before adrenalectomy	After adrenalectomy
**17-Hydroxyprogesterone, serum**	30-270 ng/dL (1.0-8.2 nmol/L)	3600 ng/dL (108.9 nmol/L)	Below detection limit
**Androstenedione, serum**	<210 ng/dL (<7.4 nmol/L)	1310 ng/dL (45.9 nmol/L)	30 ng/dL (1.1 nmol/L)
**Testosterone, serum**	26-122 ng/dL (0.9-4.2 nmol/L)	350 ng/dL (12.1 nmol/L)	15 ng/dL (0.5 nmol/L)
**DHEA-S**	0.3-2.1 µg/mL (0.8-5.7 µmol/L)	0.6 µg/mL (1.6 µmol/L)	<0.1 µg/mL (<0.3 µmol/L)
**ACTH**	4-61 pg/mL (0.9-13.4 pmol/L)	52 pg/mL (11.4 pmol/L)	11 pg/mL (2.4 pmol/L)
**Sodium**	135-145 mmol/L	144 mmol/L	140 mmol/L
**Potassium**	3.5-5.1 mmol/L	4.5 mmol/L	4.3 mmol/L
**Total cholesterol**	<200 mg/dL (<5.2 mmol/L)	209 mg/dL (5.4 mmol/L)	280 mg/dL (7.2 mmol/L)
**LDL cholesterol**	<116 mg/dL (<3.0 mmol/L)	146 mg/dL (3.8 mmol/L)	190 mg/dL (4.9 mmol/L)
**HDL cholesterol**	>48 mg/dL (>1.2 mmol/L)	43 mg/dL (1.1 mmol/L)	58 mg/dL (1.5 mmol/L)
**Fasting glucose**	60-99 mg/dL (3.3-5.5 mmol/L)	85 mg/dL (4.7 mmol/L)	94 mg/dL (6.2 mmol/L)
**HbA1c**	<5.7% (<39 mmol/mol)	5.6% (38 mmol/mol)	5.8% (40 mmol/mol)
**Total leukocyte count**	4.0-10.4 G/L (4.0-10.4 × 10^9^/L)	8.82 G/L (8.82 × 10^9^/L)	7.88 G/L (7.88 × 10^9^/L)
**Neutrophils**	1.91-7.37 G/L (1.91-7.37 × 10^9^/L)	n/a	5.50 G/L (5.50 × 10^9^/L)
**Lymphocytes**	1.22-3.56 G/L (1.22-3.56 × 10^9^/L)	n/a	1.57 G/L (1.57 × 10^9^/L)
**AST**	<34 U/L	17 U/L	29 U/L
**ALT**	<34 U/L	19 U/L	32 U/L
**GGT**	<39 U/L	21 U/L	31 U/L
**ALP**	<109 U/L	55 U/L	62 U/L

ALP, alkaline phosphatase; ALT, alanine aminotransferase; AST, aspartate aminotransferase; DHEA-S, dehydroepiandrosterone sulfate; GGT, gamma-glutamyl transferase; HbA1c, hemoglobin A1c; HDL, high-density lipoprotein; LDL, low-density lipoprotein.

An abdominal computed tomography scan showed large septate bilateral tumors intra- and retroperitoneally without calcifications or free fluid (Fig. 1). The left-sided tumor had reached a size of more than 20 cm and displaced the upper abdominal organs. The left kidney was displaced caudally, and the uterus to the right side. The computed tomography-guided biopsy revealed adrenal cortical tissue with regular differentiation and fat tissue, most likely consistent with a myelolipoma. There was no evidence for malignancy.

**Figure 1:**
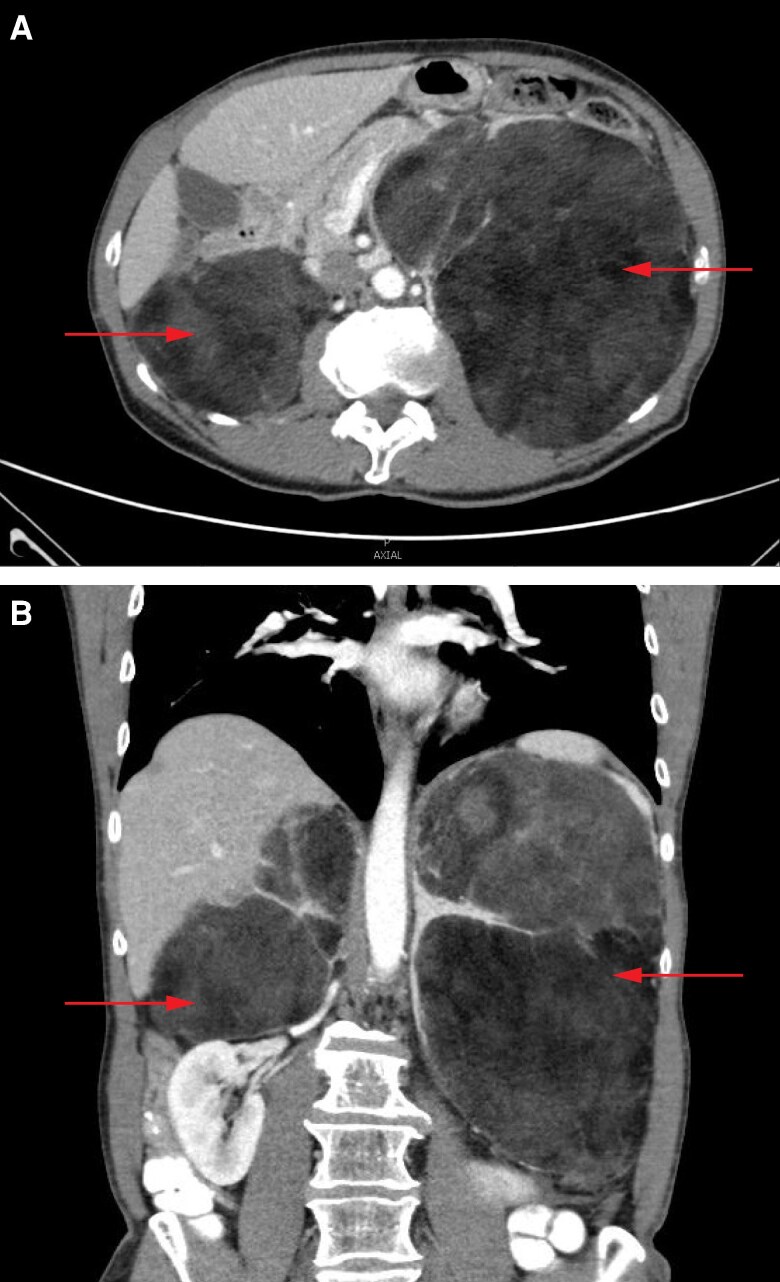
Axial (A) and coronal view (B) of the computed tomography scan demonstrating giant bilateral masses (red arrows) with the characteristic of adrenal myelolipomas.

## Treatment

Due to the giant size of the tumors and the increased risk of hemorrhage, open bilateral adrenalectomy was performed. The surgery proceeded without complications, and the tumors were completely removed. The left mass measured 24.5 cm × 20.5 cm × 9.7 cm and weighed 2230 g (Fig. 2A). The right mass measured 14.5 cm × 11.6 cm × 6.5 cm and weighed 501 g (Fig. 2B). Histological examination confirmed myelolipomas with no evidence of malignancy. The left-sided lesion predominantly consisted of extensive lipomatous tissue component with scattered areas of myeloid tissue, nodular hyperplastic adrenal cortex tissue, peripheric dense areas of residual adrenal tissue, and dense myeloid tissue (Fig. 3). Immunohistochemistry showed positive reactivity for steroidogenic factor 1. Additionally, an adrenal cortical adenoma measuring 2.5 cm was identified within the left-sided myelolipoma. The right-sided tumor demonstrated a classic myelolipoma with an extensive lipomatous tissue component and scattered areas of myeloid tissue.

**Figure 2:**
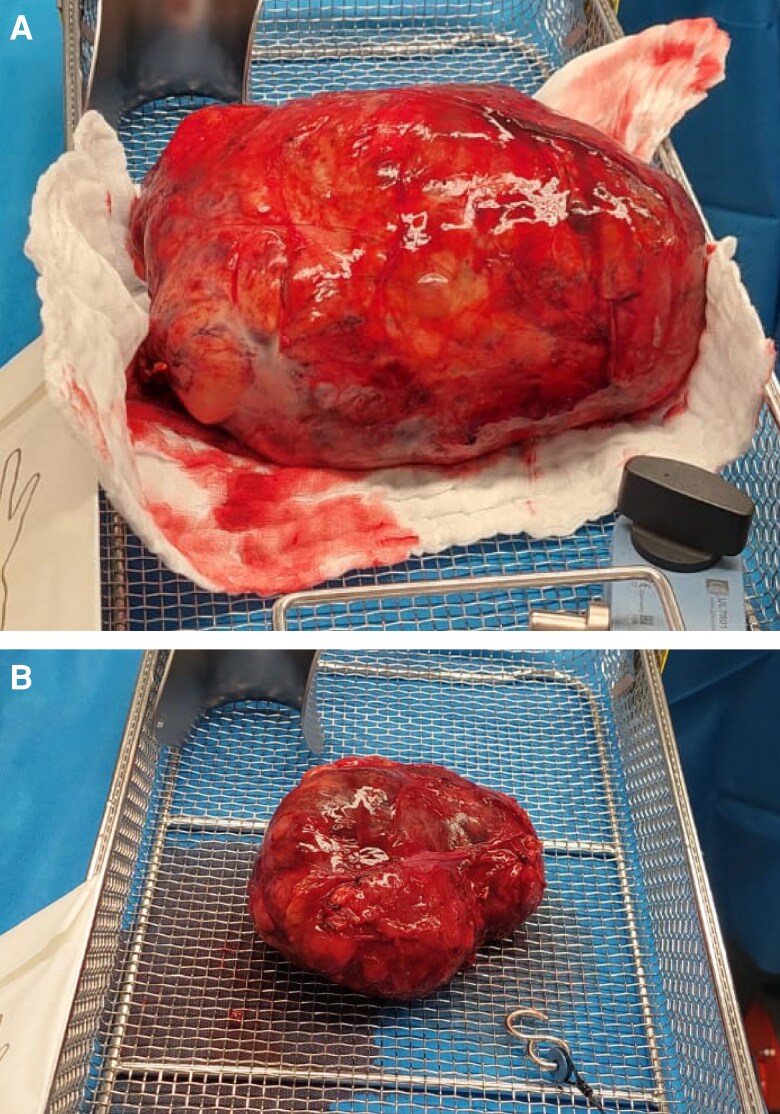
Surgical pathology of (A) left myelolipoma (size: 24.5 cm × 20.5 cm × 9.7 cm; weight: 2230 g) and (B) right myelolipoma (size: 14.5 cm × 11.6 cm × 6.5 cm; weight: 501 g).

**Figure 3:**
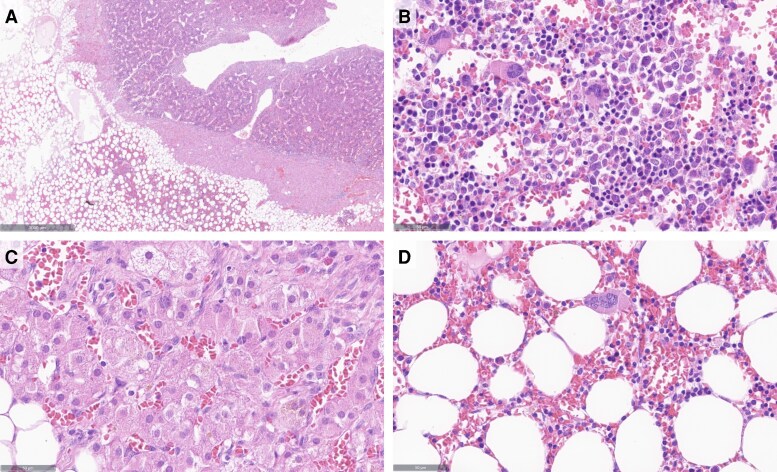
Dense area of the tumor of the left side (A) (10×, hematoxylin and eosin), showing dense myeloid tissue in trabecular organization with all 3 lineages (B), residual adrenal tissue (C), and lipomatous tissue with scattered and less dense myeloid tissue (D) (B-D, 400×, hematoxylin and eosin).

## Outcome and follow-up

The patient continued receiving lifelong glucocorticoid and mineralocorticoid replacement therapy with a total daily dose of 25 mg hydrocortisone (= 16 mg/m^2^/day) and 0.05 mg fludrocortisone (see hormonal control after bilateral adrenalectomy in Table 1). Since the bilateral adrenalectomy, no adrenal crises have occurred, and the patient maintains regular follow-ups in our clinic for endocrinology. Symptoms of hyperandrogenism gradually improved (ie, hirsutism and scalp hair loss have decreased).

## Discussion

Our case illustrates the development of giant bilateral adrenal myelolipomas in a patient with untreated classic CAH over more than 30 years. Notably, during this prolonged period, the patient neither experienced adrenal crises nor reported fatigue or impairment in daily functioning.

Adrenal myelolipomas are benign, nonfunctioning, and typically slow-growing tumors; however, increasing tumor size may lead to symptoms such as abdominal or flank pain, nausea, or vomiting due to mass effect and compression of adjacent structures [8, 9]. Individuals with CAH have a markedly increased risk of developing these tumors (10.1%) compared with the general population (0.3%) [7], and conversely, approximately 10% of all diagnosed myelolipomas are associated with CAH [8]. This strong association underscores the importance of considering underlying CAH in patients presenting with adrenal myelolipomas, particularly in the presence of bilateral lesions. This is especially relevant for giant adrenal myelolipomas (>10 cm), which are exceedingly rare in the general population [8] but occur more frequently in the context of CAH. Indeed, tumor sizes exceeding 30 × 25 × 20 cm and weights greater than 4000 g have been reported in patients with CAH [12, 13].

While our patient remained asymptomatic despite the substantial tumor burden, surgical resection is generally recommended for giant myelolipomas due to the increased risk of spontaneous rupture and hemorrhage [5].

In line with our case, previous studies report that up to 93.5% of patients with CAH and adrenal myelolipomas were poorly treated, untreated, or undiagnosed [8]. This observation supports a central role of chronically elevated ACTH levels as a key mechanistic driver in the development of adrenal myelolipomas in CAH [5]. It may also explain why these tumors occur more frequently bilaterally in patients with CAH compared with the general population. ACTH is a well-established trophic factor for the adrenal cortex, and sustained elevation in poorly treated or untreated CAH leads to the characteristic adrenal hyperplasia [5]. Consistent with this, we and others have demonstrated a positive correlation between markers of disease control and adrenal volume [6, 14].

Furthermore, some myelolipomas in patients with CAH have been shown not only to overexpress the melanocortin 2 receptor but also the androgen receptor, suggesting that both chronic ACTH stimulation and androgen excess, as in our case, may contribute to tumor pathogenesis [15].

Interestingly, some patients with CAH, despite harboring classic genotypes, do not experience adrenal crises despite biochemical adrenal insufficiency. The compound heterozygous genotype consisting of *CYP21A2* c.293-13C > G and a deletion found in our patient is generally expected to result most commonly in the salt-wasting phenotype, although some phenotypic variability has been reported for the intron 2 splice variant [16], and some patients do not need mineralocorticoid replacement during adulthood.

This phenomenon may partly be explained by the residual glucocorticoid activity of certain adrenal steroid precursors. In particular, 21-deoxycortisol and 11-deoxycorticosterone have been shown to activate the glucocorticoid receptor (GR) in vitro, whereas other precursors such as 17-OHP and progesterone exhibit minimal or negligible GR activity [17, 18]. When some mineralocorticoid capacity is preserved, ACTH-driven adrenal overstimulation may be sufficient to generate adequate amounts of 11-deoxycorticosterone and, to a lesser extent, aldosterone to activate the mineralocorticoid receptor, thereby potentially preventing adrenal crises. As no rechallenge without fludrocortisone was performed in our patient to assess mineralocorticoid demand, conclusions regarding this aspect remain speculative.

In the context of longstanding poor disease control or, as in our case, absence of treatment, chronic ACTH stimulation may lead to adrenal hyperplasia and markedly increased production of steroid precursors at levels that a normal adrenal gland would not be capable of producing. It is therefore conceivable that sustained high circulating concentrations of weak GR agonists may provide sufficient partial glucocorticoid activity, potentially mitigating the clinical manifestations of cortisol deficiency. This mechanism may contribute to the absence of adrenal crises and could explain why our patient was able to maintain daily functioning without glucocorticoid replacement therapy. Similarly, a previously reported case of late diagnosis of classic CAH with bilateral myelolipomas supports the notion that both findings may represent manifestations of longstanding adrenal overstimulation [19].

In conclusion, our case of giant bilateral myelolipomas in a female patient with classic 21-OHD is consistent with previous publications. It further supports a potential association between chronic ACTH exposure, androgen stimulation, and the growth of myelolipomas in untreated or poorly controlled CAH that is also reflected by the absence of clinical signs of adrenal insufficiency. Our case also illustrates the long-term consequences of untreated 21-OHD and the necessity and importance of ongoing multidisciplinary management.

## Learning points

- Adrenal myelolipomas are associated with CAH and should prompt evaluation for underlying CAH, particularly in the presence of bilateral or giant lesions.
- Prolonged untreated CAH can lead to significant adrenal enlargement and tumor formation, highlighting the importance of early diagnosis and adequate long-term management.
- Continuous vigilant endocrine management and long-term follow-up are essential in classic CAH to avoid or treat long-term complications.

## Contributors

N.R. and A.W. were involved in the management of the patient and manuscript submission. N.R., L.T., and M.A. were involved in the diagnosis and treatment of the patient. P.Z. was responsible for the patient's surgeries. T.B. was involved in the histopathology section and preparation of histology images. All authors reviewed and approved the final draft.

## Data Availability

Some or all datasets generated during and/or analyzed during the current study are not publicly available but are available from the corresponding author on reasonable request.
